# New Approaches and Treatment Combinations for the Management of Chronic Myeloid Leukemia

**DOI:** 10.3389/fonc.2019.00665

**Published:** 2019-08-06

**Authors:** Peter E. Westerweel, Peter A. W. te Boekhorst, Mark-David Levin, Jan J. Cornelissen

**Affiliations:** ^1^Department of Internal Medicine, Albert Schweitzer Hospital, Dordrecht, Netherlands; ^2^Department of Hematology, Erasmus Medical Center, Rotterdam, Netherlands

**Keywords:** chronic myeloid leukemia, tyrosine kinase inhibitor, interferon, chemotherapy, immunotherapy

## Abstract

Current treatment of chronic myeloid leukemia (CML) with tyrosine kinase inhibitors (TKI) is effective in many patients, but is not curative and frequently limited by intolerance or resistance. Also, treatment free remission is a novel option for CML patients and requires reaching a deep molecular remission, which is not consistently achieved with TKI monotherapy. Together, multiple unmet clinical needs remain and therefore the continued need to explore novel treatment strategies. With increasing understanding of CML biology, many options have been explored and are under investigation. This includes the use asciminib as first in class inhibitor targeting the myristoyl pocket of BCR-ABL, combination treatments with established non-TKI drugs such as interferon and drugs with novel targets relevant to CML biology such as gliptins and thiazolidinediones. Together, an overview is provided of treatment strategies in development for CML beyond current TKI monotherapy.

## Introduction

Chronic Myeloid Leukemia (CML) is a malignant myeloproliferative disease driven by the presence of the BCR-ABL1 fusion product generated as a result of the *t*(9;22) Philadelphia chromosome (Ph). Current treatment revolves around the use of Tyrosine Kinase Inhibitors (TKI) such as imatinib, which inhibit the oncogenic potential of the BCR-ABL1 protein by blocking its kinase domain. However, clinical challenges in the treatment of CML persist. Here, we aim to provide an overview of some new approaches in clinical development for the treatment of CML. We have limited this overview to chronic phase CML as accelerated and blast phase CML are clinically and biologically very distinct disease entities.

Prior to the introduction of the TKI imatinib, the main treatment options for CML included allogeneic stem cell transplantation, chemotherapy such as hydroxyureum or busulphan, and interferon. With the revolutionizing treatment potential of imatinib, approved by the FDA and EMA in 2001, the focus since then has been on optimizing the use of TKI therapy. After imatinib, second and third generation TKIs were developed: bosutinib, dasatinib, nilotinib, radotinib, and ponatinib. All these compounds target the ATP binding pocket in the kinase domain of the BCR-ABL oncoprotein, but have a different affinity in the presence of certain mutations in the kinase domain due to variations in their chemical structure. This also results in different off-target effects on other tyrosine kinases and thereby the specific toxicity profile, which is a major clinical factor as approximately half of the treatment discontinuations are due to TKI toxicity (1). Although the great majority of patients with chronic phase CML have an excellent prognosis, intolerance to currently available TKI is not uncommon and resistance to TKI therapy due to BCR-ABL mutations occurs frequently (1). CML-related death due to progression from chronic to advanced phase disease remains a reality and ranges up to 15%, depending on clinical factors at diagnosis as incorporated in e.g., the EUTOS long term survival score (ELTS) (2, 3).

Although TKI therapy results in a loss of excessive bone marrow proliferation with effective induction of clinical remission and prevention of progression to advanced phase of the disease, the treatment is presumed not to be curative. Ph+ CML leukemic stem cells are not dependent on BCR-ABL1 activity for their survival as they reside quiescently in the bone marrow. Indeed, after prolonged TKI treatment and clinical disease remission, the BCR-ABL1 fusion product remains detectable in virtually all CML patients. These low levels of residual disease are routinely quantified by using Real-Time Quantitative Polymerase Chain Reaction (RT-qPCR) for BCR-ABL1 demonstrating so-called deep molecular remissions. Although continuation of therapy was initially considered essential, recent studies have shown that ~50% of patients in sustained deep molecular remission may stop their TKI treatment without clinical relapse (4, 5). Importantly, patients showing loss of a major molecular remission with a BCR-ABL1 rising above 0.1% on the international scale, universally regain their major molecular remission upon re-initiation of their TKI. With close molecular monitoring and timely re-initiation of TKI therapy, TKI discontinuation has been proven to be safe in carefully selected patients (4, 5). Since the BCR-ABL1 fusion product remains detectable in many patients after TKI discontinuation, this condition is called “Treatment Free Remission” (TFR), reflecting an operational, but not true cure. Maintenance of low-levels of residual disease is supposed to occur through immune regulatory mechanisms supported by associations of successful TFR with KIR (Killer Cell Immunoglobulin-like Receptors) haplotypes, T-cell CD62L expression and CD56 cytotoxic T-cell numbers (4–8). Achieving TFR is now an important new treatment goal in CML for both doctors and patients (9). Successful TFR improves quality of life of patients as they become free of TKI side effects, are no longer exposed to potential late toxicity and brings substantial drug cost savings. However, only a minority of patients is eligible to try and discontinue their TKI. In clinical practice, this accumulates to 31% after 6 years of TKI treatment (1).

New treatment approaches continue to be needed to improve disease control, avoid the development of TKI resistance and progression to advanced disease, provide an alternative to current TKI therapy to mitigate TKI related toxicity, or to improve the chance of successfully discontinuing therapy to achieve a TFR.

## Combination Therapy With TKI for Chronic Phase CML

Preclinical investigations suggest an additive effect of various classic antineoplastic agents including interferon-alpha, daunorubicin, etoposide, cytarabine (cytosine arabinoside, Ara-C), if added to imatinib *in vitro* (10, 11). Several of these combinations have also been explored in clinical studies (Table 1).

**Table 1:** Overview of selected published and ongoing trials investigating (combination) treatments for chronic phase CML.

	**Published trials**	**Ongoing trials**
**TKI/interferon combinations**		
Bosutinib + pegylated interferon-alpha		NCT03831776
Dasatinib + pegylated interferon-alpha	Phase II (12)	
Imatinib + (pegylated)-interferon-alpha	Phase III (13–16)	
Nilotinib + pegylated interferon-alpha	Phase II (17)	NCT01657604
		NCT02201459
**TKI/chemotherapy combinations**		
Imatinib + hydroxyurea	Phase II (18)	
Imatinib + cytarabine	Phase III (15, 16, 19)	
Imatinib + HHT/omacetaxine	Phase II (20)	
HHT/omacetaxine alone	Phase II (20–22)	
**Novel TKI inhibitor Asciminib**		NCT03595917
		NCT02081378
		NCT03106779
**Immune modulation**		
Dendritic cell vaccinations	Phase I/II (23, 24)	
Leukemia associated antigen vaccinations	Phase I/II (25, 26)	
Immune checkpoint inhibitors		
Nivolumab + dasatinib		NCT02011945
Avelumab + various TKI		NCT02767063
**PPAR-γ** **agonists** (thiazolidinediones)	Phase II (27)	NCT02767063
**DPPIV inhibitors** (gliptins)		2017-000899-28

### TKI + Interferon

Interferon-alpha is effective as monotherapy in patients with CML and was the treatment of choice over chemotherapy in the pre-imatinib era (28, 29). Interferon-alpha exerts its antileukemic effect through a direct anti-proliferative effect specifically on CML progenitor cells (30, 31) but there may be an additional immunomodulatory mode of action (32). The clinical trial leading to the registration of imatinib was the randomized IRIS trial, which showed marked superiority of imatinib compared to interferon in combination with low-dose cytarabine (33). In the TKI era that followed, interferon monotherapy retained a limited place in clinical practice, e.g., for patients who were TKI intolerant or could not be treated with a TKI during pregnancy.

However, because of their different modes of action, exploration of the potential to combine TKI therapy with interferon soon followed. Early observations noted dose-dependent toxicity that limited the use of higher dosages of interferon cotreatment (34). However, at relatively low dosages, combination of interferon and imatinib proved better feasible and resulted in higher rates of cytogenetic and molecular remission in multiple studies. The Italian GIMEMA study group showed significantly higher complete cytogenetic remission (60 vs. 42%, *p* = 0.003) and major molecular (67 vs. 47%, *p* = 0.001) remission rates for imatinib + interferon treated patients (*n* = 76) than for patients treated with imatinib alone (*n* = 419) at 6 months of treatment. However, at 12 months, 59% of patients had discontinued interferon and this percentage increased to 87% at 24 months, which may explain the lack of difference in remission rates after 4 years follow up in the trial (13). A randomized controlled trial by the Nordic CML study group found highermajor molecular response rates at 12 months for the combination of interferon and imatinib compared to imatinib alone (82 vs. 54%, *p* = 0.002; *n* = 56 for each arm), even though 61% of patients discontinued interferon, mainly due to toxicity. No long term results have been reported (14). In line with these results, the large French SPIRIT trial observed significantly higher major molecular response rates after 12 and 24 months in the interferon-imatinib group than in the group treated with imatinib alone (57 vs. 38%, *p* < 0.001 and 64 vs. 43%; *p* = 0.006, respectively, *n* = 159 in each arm). Interestingly, the rate of deep molecular response (MR4.0) was also higher. Again, the interferon discontinuation rate was high (83%), mainly due to toxicity, despite a reduction in the interferon dose during the study (15). Finally, the even larger German CML IV trial compared imatinib treated patients (*n* = 400) with several combinations, including a study arm in which imatinib was combined with interferon (*n* = 430). Consistently, rates and depth of molecular remission were numerically better when interferon was added to imatinib, but few patients continued interferon for the long term and no statistical difference in overall survival was observed (16). Indeed, none of the aforementioned studies found a statistically significant reduction in progression to advanced phase disease or prevention of CML-related death. Taken together, combination treatment of imatinib with interferon in the upfront setting therefore does improve the speed and depth of disease remission on imatinib treatment, but without a benefit on overall survival and accompanied by significant toxicity resulting in high rates of interferon discontinuation.

Interferon has also been combined with second generation TKIs nilotinib (17) and dasatinib (12) in single arm studies with good efficacy. The combination of nilotinib with peginterferon alfa-2a at a dose of 90 μg per week, tapered to 45 μg per week after 1 month treatment was associated with a substantial incidence of adverse events (17), which is why a later study used a lower dose (NordDutchCML009 study, ClinicalTrials.gov Identifier: NCT01866553). Dasatinib combined with even lower doses of peginterferon alfa-2b (15 μg increasing to 25 μg/week) was well-tolerated (12). Together, this paved the way for phase III randomized trials combining second generation TKI with interferon, such as the German TIGER study comparing nilotinib monotherapy to a combination of nilotinib with peginterferon alpha-2b (ClinicalTrials.gov Identifier: NCT01657604) and the French PETALs study combining nilotinib and peginterferon alpha-2a (ClinicalTrials.gov Identifier: NCT02201459). No results have been published for the combination of bosutinib with interferon, but this is being investigated in the BosuPeg trial (ClinicalTrials.gov Identifier NCT03831776). The latter study uses a novel generation of mono-pegylated interferon, ropeginterferon alfa-2b, which has a longer half-life allowing biweekly administration and was markedly well-tolerated in patients with polycythemia vera (35).

A new interest in interferon came when patients previously treated with interferon were found to retain their molecular remission after imatinib discontinuation numerically more often in the STIM and A-STIM French TKI discontinuation trials, although without achieving statistical significance (36, 37). In the large Euro-SKI trial that later followed, patients who used interferon for a substantial period during prior treatment (>1.5 years), demonstrated a higher chance of durable TKI discontinuation which was proven to be statistically significant (38). However, the number of patients in this subgroup was limited and selection bias may have occurred. Nonetheless, combination therapy of a TKI with a low dose of interferon for a limited period of time prior to a TKI discontinuation attempt could be an interesting option to increase the TFR success rate. Indeed, such a strategy is a provisional experimental arm in the French ACTIW study, in which various combination treatments prior to TKI discontinuation are explored (ClinicalTrials.gov Identifier: NCT02767063).

Across the interferon trials, the various pegylated forms of interferon have been most promising, are better tolerated and more practical for the patient than non-pegylated interferon. No specific subtype has been proven to be superior as no head to head comparisons between pegylated interferon variants are available in the context of CML. A major issue for the future development of interferon as adjunctive therapy in CML is the availability of these pegylated interferon formulations because pharmaceutical companies have recently announced that they may be discontinuing their production as the primary market, the treatment of hepatitis C, is diminishing.

### TKI + Chemotherapy

#### Hydroxyurea

Hydroxyurea is a classic chemotherapeutic drug, which was used to limit leucocyte proliferation before the introduction of interferon and TKIs. Hydroxyurea is currently still widely used as temporary treatment to mitigate leukocytosis in *de novo* CML, either as short-term monotherapy prior to TKI initiation, e.g., while awaiting confirmatory diagnostic testing, or in addition to TKI therapy to accelerate the rate of cytoreduction. This may be useful in rare cases of symptomatic hyperleukocytosis, but a randomized phase II study showed no benefit of hydroxyurea as standard additive to imatinib in newly diagnosed patients for achieving remissions in the course of the disease (18). Also from preclinical investigations, hydroxyurea was not found to be synergistic to imatinib and even antagonized some of its antiproliferative effect (10).

#### Cytarabine

Adding an intermediate dose cytarabine to imatinib for the upfront treatment of chronic phase CML proved feasible in clinical practice (39). Initial studies suggested a beneficial effect of adding cytarabine to imatinib (40–42). However, no beneficial effect was observed in later randomized phase III studies, while it did increase adverse event rates (15, 16, 19). Although some differences in dosing may explain the discrepant findings, the addition of low to intermediate doses of cytarabine to TKI in treatment of chronic phase CML is unlikely to be further pursued.

#### Homoharringtonine and Omacetaxin

Homoharringtonine (HHT), a plant alkaloid derived from Cephalotaxus species, and a synthetic formulation of HHT called omacetaxine, have activity against CML cells through inhibition of ribosomal protein translation. It is active as monotherapy, delivered first intravenously followed by twice daily subcutaneous injections (21, 22). Particularly interesting is the activity of omacetaxin in CML patients harboring the T315I mutation in which a complete hematological response was observed in 77% of the patients, although deep and durable remissions were rare (43). Omacetaxine is registered in the United States for the treatment of patients with CML in chronic of accelerated phase after failure of 2 or more TKIs (44). The EMA application was not pursued by the company, so it is not available in Europe.

Omacetaxin has been investigated in combination with imatinib in a small study including CML patients in chronic phase with TKI resistance or patients with advanced disease, in which overall response rate was 40% after 4 months (20).

### Novel Allosteric BCR-ABL Inhibitor Asciminib

Apart from the ATP binding pocket in the kinase domain, the BCR-ABL oncoprotein has a second domain suitable for pharmacological targeting. This domain is characterized by a myristoyl binding pocket and serves as an autoinhibitory site for the wildtype ABL protein. At its N-terminal end, the ABL protein has a myristoyl group, which binds to the myristoyl pocket resulting in conformational change and thereby allosteric “autoinhibition” of kinase activity. In the BCR-ABL oncoprotein, this myristoyl group is no longer present as it is lost upon fusion of BCR at the N-terminal end of ABL. This is why BCR-ABL is constitutively active. Novel so called allosteric inhibitors targeting this myristoyl pocket have shown *in vitro* and *in vivo* TKI activity (45). The compound most maturely developed is ABL001, recently renamed asciminib.

Asciminib is active in inhibiting BCR-ABL positive CML cell lines and primary hematopoietic cells from CML patients (45). Its activity is, as would be expected, not influenced by mutations coding for variations in the ATP binding pocket, including e.g., the T315I mutation, known to cause resistance to all currently available TKIs except ponatinib. Importantly, asciminib has no activity against non-BCR-ABL harboring cell lines, supporting the highly selective action against BCR-ABL without off-target inhibition of other tyrosine kinases. In an animal xenograft model, asciminib inhibited the expansion of an infused KCL-22 CML cell line, even after acquiring resistance to the TKI nilotinib *in vivo*. However, acquired resistance to asciminib has also been observed in this model in case mutations occur in the myristoyl binding site. In this case, sequential treatment with nilotinib was temporarily effective until a second mutation occurred in the ATP binding site. Interestingly, no mutational escape was noted if nilotinib was combined with asciminib and in fact, the leukemic clone was completely eradicated as combined treatment could be stopped without recurrence (45). These promising *in vitro* observations and *in vivo* animal experiments suggest applicability in clinical practice for both monotherapy and combination therapy.

Asciminib has been tested in a phase I trial for patients with BCR-ABL positive CML in chronic and advanced phase and in Ph-positive ALL (ClinicalTrials.gov Identifier: NCT02081378). A specific cohort was included for patients harboring the T315I mutation. In this phase I trial, both asciminib monotherapy and combination therapy with imatinib, dasatinib, and nilotinib are being investigated. To date, only the results of asciminib monotherapy in the phase I trial have been publicly presented and showed significant and durable activity in this patient cohort with overall good tolerance (46). Toxicity was mostly CTCAE grade 1/2. Observed CTCAE Grade 3/4 toxicity included lipase increase and hematological toxicity.

Several studies are running to further explore the clinical potential of asciminib. A phase I trial is investigating a combination of dasatinib, asciminib, and prednisone for Ph+ ALL or CML in lymphoid blast crisis (ClinicalTrials.gov Identifier: NCT03595917). A phase II study is investigating adding asciminib to imatinib in patients with CML treated with imatinib for at least 2 years who have failed to achieve a deep molecular remission at the MR4.0 level (ClinicalTrials.gov Identifier: NCT03578367). Optimizing the molecular response to MR4.0 or MR4.5 would potentially enable patients to stop TKI. One phase III trial is running, in which asciminib monotherapy is randomized against bosutinib for the treatment of CML patients with resistance or intolerance to at least two TKIs targeting the kinase domain (ClinicalTrials.gov identifier NCT03106779). Other studies with asciminib are in preparation and are likely to impact the treatment of CML in the future.

## Additional Pathways

Over the years, much has become clear about the biology of CML and CML stem cells, which has led to the identification of many pathways that may be potentially targeted. For a very comprehensive review see the paper of Massimino et al. (47). Below, we discuss a selection of the most relevant explorations.

### Immune Modulation

In general, CML is well-known as a disease sensitive to immunological control, as evidenced by the fact that immune responses against CML-specific and CML-associated antigens such as BCR-ABL1, proteinase-3, and WT-1 can be detected in CML patients (48) and donor lymphocyte infusions are able to induce long-lasting remissions in relapsed CML patients after allogeneic SCT (49).

Several small studies have investigated the potential to induce an anti-leukemic vaccination response in CML patients. A first strategy is to vaccinate using *ex vivo* generated autologous dendritic cells. This has been shown to indeed induce a CML-specific T-cell response with proliferative capacity after injection of *ex-vivo* generated autologous dendritic cells (23, 24). Another option is to vaccinate patients using leukemia associated antigens. Injection of BCR-ABL derived peptides was found to be safe and feasible and indeed induced a T-cell response in the majority of patients (25, 26). In these single-arm studies, some patients showed a reduction of cytogenetic and/or molecular depth of response implying clinical efficacy. But as these were single arm studies, this cannot be determined with certainty. Prospective randomized trials are needed, but even though the initial observations were made more than a decade back, little progress has been made to move toward randomized controlled phase II or III trials.

From a theoretical point of view, treatment with immune checkpoint inhibitors could potentially increase immunoreactivity against leukemic cells in CML. Indeed, translational work suggests that PD-L1 upregulation is an immunological escape mechanism for CML cells (50). Few clinical observations have been published or presented, but a phase 1 trial using PD-L1 inhibitor nivolumab in combination with dasatinib has recently been completed with results pending (ClinicalTrials.gov Identifier: NCT02011945) and PD-L1 inhibitor avelumab is under investigation for CML patients in the ACTIW trial (ClinicalTrials.gov Identifier: NCT02767063).

### Thiazolidinediones (PPAR-γ Agonists)

Thiazolidinediones are peroxisome proliferator-activated receptor gamma (PPAR-γ) agonists, which are under investigation for use as synergizing co-treatment together with TKI in CML. Thiazolidinediones are already used in clinical practice for the treatment of diabetes, where they act as transcription factors that increase tissue sensitivity to insulin. *In vitro*, PPAR-γ agonist pioglitazone induced CML cells to exit quiescence, which in turn sensitizes them to the effects of imatinib. PPAR-γ agonists serve as transcription factors that downregulate STAT5 and its targets HIF2α and CITED2, which are overexpressed in CML stem cells (51). After promising results in a case series (51) and a single arm phase 2 study (27), combination treatment of PPAR-γ agonist pioglitazone with imatinib is now under prospective randomized investigation (ClinicalTrials.gov Identifier: NCT02767063).

### Gliptins (DPPIV Inhibitors)

By sheer coincidence, another drug class used for the treatment of diabetes is under investigation for use as co-treatment in CML: the gliptins. Ph+CD34+ CML cells were found to aberrantly express dipeptidylpeptidase IV (DPPIV), also known as CD26 (52). Membrane-bound DPPIV is a cleaving protease that inactivates SDF1, resulting in deregulation of the hematopoietic niche. DPPIV is not found on hematopoietic CD34+ cells from patients with other myeloid neoplasms or healthy controls (52). DPPIV also inactivates various other cytokines such as incretins, which regulate post-prandial insulin secretion. This is why DPPIV inhibitors are used for the treatment of diabetes, notably without causing hypoglycemia in non-diabetic individuals. In CML, gliptins inhibit aberrantly expressed membrane-bound DPPIV, thereby restoring the disrupted SDF1 gradient resulting in normalization of the interaction within the hematopoietic niche. In anecdotal CML patients with coincidental diabetes, the initiation of treatment with a gliptin was associated in time with a deepening of molecular remission (52). A phase 1/2 study combining TKI nilotinib with vildagliptin as pre-treatment for a TKI discontinuation attempt is currently recruiting (clinicaltrialsregister.eu. EudraCT: 2017-000899-28).

## Conclusion

With the introduction of TKI therapy now nearly 20 years ago, the landscape of CML treatment has fundamentally changed and the general prognosis of patients presenting in chronic phase of the disease is excellent. However, TKI therapy is not curative and long-term exposure to TKI is associated with chronic side effects, potential health hazards and a financial burden for health care systems. TKI intolerance is a frequent clinical problem. Also, some patients develop TKI resistance resulting in progression to advanced phase and ultimately CML related death. Treatment free remission is a novel option for CML patients, but achievable for only a small minority of patients. Together, multiple unmet clinical needs remain, which clarifies the continued need to explore novel treatment strategies. With increasing understanding of CML biology, many options have been explored. This may include use of the novel class of targeted BCR-ABL1 inhibitors targeting the myristoyl pocket of BCR-ABL, combination treatments with established non-TKI drugs such as interferon or other drugs with novel targets relevant to CML biology.

## Author Contributions

PW performed a literature search and wrote the initial draft, which was revised and amended by PtB, ML, and JC. All authors read and approved the final version of the manuscript.

### Conflict of Interest Statement

The authors declare that the research was conducted in the absence of any commercial or financial relationships that could be construed as a potential conflict of interest.

## Abbreviations

ABL: Abelson tyrosine kinase protein
ALL: Acute Lymphatic Leukemia
Ara-C: Cytosine Arabinoside
BCR-ABL: Breakpoint Cluster Region—Abelson fusion product
CITED2: Cbp/p300-Interacting Transactivator with Glu/Asp-rich carboxy-terminal Domain 2
CML: Chronic Myeloid Leukemia
CTCAE: Common Terminology Criteria for Adverse Events
DPPIV: DiPeptidylPeptidase IV
ELTS: EUTOS Long Term Survival Score
EudraCT: European Union Drug Regulating Authorities Clinical Trials
GIMEMA: Gruppo Italiano Malattie EMatologiche dell'Adulto (Italian collaborative group for adult hematological diseases)
HHT: Homoharringtonine
HIF: Hypoxia Inducible Factor
KIR: Killer cell Immunoglobulin-like Receptors
PD-L1: Programmed death-ligand 1
Ph: Philadelphia Chromosome
PPAR- γ: Peroxisome Proliferator-Activated Receptor Gamma
RT-qPCR: Real-Time Quantitative Polymerase Chain Reaction
STAT5: Signal Transducer and Activator of Transcription 5
TFR: Treatment Free Remission
TKI: Tyrosine Kinase Inhibitor(s).

## References

[1] Geelen IGP Thielen N Janssen JJWM Hoogendoorn M Roosma TJA Willemsen SP. Treatment outcome in a population-based, 'real-world' cohort of patients with chronic myeloid leukemia. Haematologica. (2017) 102:1842–9. 10.3324/haematol.2017.174953 28860339 PMC5664388

[2] Pfirrmann M Baccarani M Saussele S Guilhot J Cervantes F Ossenkoppele G. Prognosis of long-term survival considering disease-specific death in patients with chronic myeloid leukemia. Leukemia. (2016) 30:48–56. 10.1038/leu.2015.261 26416462

[3] Geelen IGP Sandin F Thielen N Janssen JJWM Hoogendoorn M Visser O. Validation of the EUTOS long-term survival score in a recent independent cohort of real world CML patients. Leukemia. (2018) 32:2299–303. 10.1038/s41375-018-0136-7 29743721

[4] Hughes TP Ross DM. Moving treatment-free remission into mainstream clinical practice in CML. Blood. (2016) 128:17–23. 10.1182/blood-2016-01-694265 27013442

[5] Saussele S Richter J Hochhaus A Mahon FX. The concept of treatment-free remission in chronic myeloid leukemia. Leukemia. (2016) 30:1638–47. 10.1038/leu.2016.115 27133824 PMC4980559

[6] Caocci G Martino B Greco M Abruzzese E Trawinska MM Lai S. Killer immunoglobulin-like receptors can predict TKI treatment-free remission in chronic myeloid leukemia patients. Exp Hematol. (2015) 43:1015–8.e1. 10.1016/j.exphem.2015.08.004 26306453

[7] Ilander M Olsson-Stromberg U Schlums H Guilhot J Bruck O Lahteenmaki H. Increased proportion of mature NK cells is associated with successful imatinib discontinuation in chronic myeloid leukemia. Leukemia. (2016) 31:1108–16. 10.1038/leu.2016.360 27890936 PMC5420794

[8] Schutz C Inselmann S Saussele S Dietz CT Mu Ller MC Eigendorff E. Expression of the CTLA-4 ligand CD86 on plasmacytoid dendritic cells (pDC) predicts risk of disease recurrence after treatment discontinuation in CML. Leukemia. (2017) 31:829–36. 10.1038/leu.2017.9 28074067

[9] Saglio G Sharf G Almeida A Bogdanovic A Bombaci F Cugurovic J. Considerations for treatment-free remission in patients with chronic myeloid leukemia: a joint patient-physician perspective. Clin Lymphoma Myeloma Leuk. (2018) 18:375–9. 10.1016/j.clml.2018.04.005 29753691

[10] Thiesing JT Ohno-Jones S Kolibaba KS Druker BJ. Efficacy of STI571, an abl tyrosine kinase inhibitor, in conjunction with other antileukemic agents against bcr-abl-positive cells. Blood. (2000) 96:3195–9. Available online at: http://www.bloodjournal.org/content/96/9/3195 11050003

[11] Topaly J Zeller WJ Fruehauf S. Synergistic activity of the new ABL-specific tyrosine kinase inhibitor STI571 and chemotherapeutic drugs on BCR-ABL-positive chronic myelogenous leukemia cells. Leukemia. (2001) 15:342–7. 10.1038/sj.leu.2402041 11237055

[12] Hjorth-Hansen H Stentoft J Richter J Koskenvesa P Hoglund M Dreimane A. Safety and efficacy of the combination of pegylated interferon-alpha2b and dasatinib in newly diagnosed chronic-phase chronic myeloid leukemia patients. Leukemia. (2016) 30:1853–60. 10.1038/leu.2016.121 27133821

[13] Palandri F Castagnetti F Iacobucci I Martinelli G Amabile M Gugliotta G. The response to imatinib and interferon-alpha is more rapid than the response to imatinib alone: a retrospective analysis of 495 Philadelphia-positive chronic myeloid leukemia patients in early chronic phase. Haematologica. (2010) 95:1415–9. 10.3324/haematol.2009.021246 20305139 PMC2913092

[14] Simonsson B Gedde-Dahl T Markevarn B Remes K Stentoft J Almqvist A. Combination of pegylated IFN-alpha2b with imatinib increases molecular response rates in patients with low- or intermediate-risk chronic myeloid leukemia. Blood. (2011) 118:3228–35. 10.1182/blood-2011-02-336685 21685374

[15] Preudhomme C Guilhot J Nicolini FE Guerci-Bresler A Rigal-Huguet F Maloisel F. Imatinib plus peginterferon alfa-2a in chronic myeloid leukemia. N Engl J Med. (2010) 363:2511–21. 10.1056/NEJMoa1004095 21175313

[16] Hehlmann R Lauseker M Saussele S Pfirrmann M Krause S Kolb HJ. Assessment of imatinib as first-line treatment of chronic myeloid leukemia: 10-year survival results of the randomized CML study IV and impact of non-CML determinants. Leukemia. (2017) 31:2398–406. 10.1038/leu.2017.253 28804124 PMC5668495

[17] Nicolini FE Etienne G Dubruille V Roy L Huguet F Legros L. Nilotinib and peginterferon alfa-2a for newly diagnosed chronic-phase chronic myeloid leukaemia (NiloPeg): a multicentre, non-randomised, open-label phase 2 study. Lancet Haematol. (2015) 2:e37–46. 10.1016/S2352-3026(14)00027-1 26687426

[18] Lange T Krahl R Grünhagen Uv Al-Ali HK Schwarzer A Jentsch-Ullrich K Imatinib (IM) in combination with Hydroxyurea in patients with CML1st CP does not increase molecular response at 18 months compared to IM alone. Final results of the OSHO CML2004 study. Oncol Res Treat. (2017) 40:121–2. 10.1038/sj.leu.2402041

[19] Thielen N van der Holt B Verhoef GE Ammerlaan RA Sonneveld P Janssen JJ. High-dose imatinib versus high-dose imatinib in combination with intermediate-dose cytarabine in patients with first chronic phase myeloid leukemia: a randomized phase III trial of the Dutch-Belgian HOVON study group. Ann Hematol. (2013) 92:1049–56. 10.1007/s00277-013-1730-4 23572137

[20] Maiti A Cortes J Ferrajoli A Estrov Z Borthakur G Garcia-Manero G. Phase II trial of homoharringtonine with imatinib in chronic, accelerated, and blast phase chronic myeloid leukemia. Leuk Lymphoma. (2017) 58:1–6. 10.1080/10428194.2017.1283030 28278723 PMC5478443

[21] Quintas-Cardama A Kantarjian H Garcia-Manero G O'Brien S Faderl S Estrov Z. Phase I/II study of subcutaneous homoharringtonine in patients with chronic myeloid leukemia who have failed prior therapy. Cancer. (2007) 109:248–55. 10.1002/cncr.22398 17154172

[22] Cortes JE Kantarjian HM Rea D Wetzler M Lipton JH Akard L. Final analysis of the efficacy and safety of omacetaxine mepesuccinate in patients with chronic- or accelerated-phase chronic myeloid leukemia: Results with 24 months of follow-up. Cancer. (2015) 121:1637–44. 10.1002/cncr.29240 25586015 PMC5650096

[23] Litzow MR Dietz AB Bulur PA Butler GW Gastineau DA Hoering A. Testing the safety of clinical-grade mature autologous myeloid DC in a phase I clinical immunotherapy trial of CML. Cytotherapy. (2006) 8:290–8. 10.1080/14653240600735743 16793737

[24] Westermann J Kopp J van Lessen A Hecker AC Baskaynak G le Coutre P. Vaccination with autologous non-irradiated dendritic cells in patients with bcr/abl+ chronic myeloid leukaemia. Br J Haematol. (2007) 137:297–306. 10.1111/j.1365-2141.2007.06547.x 17408402

[25] Cathcart K Pinilla-Ibarz J Korontsvit T Schwartz J Zakhaleva V Papadopoulos EB. A multivalent bcr-abl fusion peptide vaccination trial in patients with chronic myeloid leukemia. Blood. (2004) 103:1037–42. 10.1182/blood-2003-03-0954 14504104

[26] Rojas JM Knight K Wang L Clark RE. Clinical evaluation of BCR-ABL peptide immunisation in chronic myeloid leukaemia: results of the EPIC study. Leukemia. (2007) 21:2287–95. 10.1038/sj.leu.2404858 17637811

[27] Rousselot P Prost S Guilhot J Roy L Etienne G Legros L. Pioglitazone together with imatinib in chronic myeloid leukemia: a proof of concept study. Cancer. (2017) 123:1791–9. 10.1002/cncr.30490 28026860 PMC5434901

[28] Talpaz M Kantarjian H Kurzrock R Trujillo JM Gutterman JU. Interferon-alpha produces sustained cytogenetic responses in chronic myelogenous leukemia. Philadelphia chromosome-positive patients. Ann Intern Med. (1991) 114:532–8. 2001086 10.7326/0003-4819-114-7-532

[29] Kantarjian HM Smith TL O'Brien S Beran M Pierce S Talpaz M. Prolonged survival in chronic myelogenous leukemia after cytogenetic response to interferon-alpha therapy. The Leukemia Service. Ann Intern Med. (1995) 122:254–61. 7825760 10.7326/0003-4819-122-4-199502150-00003

[30] Cornelissen JJ Ploemacher RE Wognum BW Borsboom A Kluin-Nelemans HC Hagemeijer A. An *in vitro* model for cytogenetic conversion in CML. Interferon-alpha preferentially inhibits the outgrowth of malignant stem cells preserved in long-term culture. J Clin Invest. (1998) 102:976–83. 10.1172/JCI2366 9727066 PMC508963

[31] Gordon MY Marley SB Lewis JL Davidson RJ Nguyen DX Grand FH. Treatment with interferon-alpha preferentially reduces the capacity for amplification of granulocyte-macrophage progenitors (CFU-GM) from patients with chronic myeloid leukemia but spares normal CFU-GM. J Clin Invest. (1998) 102:710–5. 10.1172/JCI3094 9710439 PMC508933

[32] Guilhot F Roy L Guilhot J Millot F. Interferon therapy in chronic myelogenous leukemia. Hematol Oncol Clin North Am. (2004) 18:585–603, viii. 10.1016/j.hoc.2004.03.002 15271394

[33] O'Brien SG Guilhot F Larson RA Gathmann I Baccarani M Cervantes F. Imatinib compared with interferon and low-dose cytarabine for newly diagnosed chronic-phase chronic myeloid leukemia. N Engl J Med. (2003) 348:994–1004. 10.1056/NEJMoa022457 12637609

[34] Baccarani M Martinelli G Rosti G Trabacchi E Testoni N Bassi S. Imatinib and pegylated human recombinant interferon-alpha2b in early chronic-phase chronic myeloid leukemia. Blood. (2004) 104:4245–51. 10.1182/blood-2004-03-0826 15319292

[35] Gisslinger H Zagrijtschuk O Buxhofer-Ausch V Thaler J Schloegl E Gastl GA. Ropeginterferon alfa-2b, a novel IFNalpha-2b, induces high response rates with low toxicity in patients with polycythemia vera. Blood. (2015) 126:1762–9. 10.1182/blood-2015-04-637280 26261238 PMC4608390

[36] Mahon FX Rea D Guilhot J Guilhot F Huguet F Nicolini F. Discontinuation of imatinib in patients with chronic myeloid leukaemia who have maintained complete molecular remission for at least 2 years: the prospective, multicentre Stop Imatinib (STIM) trial. Lancet Oncol. (2010) 11:1029–35. 10.1016/S1470-2045(10)70233-3 20965785

[37] Rousselot P Charbonnier A Cony-Makhoul P Agape P Nicolini FE Varet B. Loss of major molecular response as a trigger for restarting tyrosine kinase inhibitor therapy in patients with chronic-phase chronic myelogenous leukemia who have stopped imatinib after durable undetectable disease. J Clin Oncol. (2014) 32:424–30. 10.1200/JCO.2012.48.5797 24323036

[38] Saussele S Richter J Guilhot J Gruber FX Hjorth-Hansen H Almeida A. Discontinuation of tyrosine kinase inhibitor therapy in chronic myeloid leukaemia (EURO-SKI): a prespecified interim analysis of a prospective, multicentre, non-randomised, trial. Lancet Oncol. (2018) 19:747–57. 29735299 10.1016/S1470-2045(18)30192-X

[39] Deenik W van der Holt B Verhoef GE Smit WM Kersten MJ Kluin-Nelemans HC. Dose-finding study of imatinib in combination with intravenous cytarabine: feasibility in newly diagnosed patients with chronic myeloid leukemia. Blood. (2008) 111:2581–8. 10.1182/blood-2007-08-107482 18172005

[40] Gardembas M Rousselot P Tulliez M Vigier M Buzyn A Rigal-Huguet F. Results of a prospective phase 2 study combining imatinib mesylate and cytarabine for the treatment of Philadelphia-positive patients with chronic myelogenous leukemia in chronic phase. Blood. (2003) 102:4298–305. 10.1182/blood-2003-04-1010 12933584

[41] Hurtado-Monroy R Vargas-Viveros P Candelaria M Cervera E Cruz J Gutierrez O Imatinib compared with imatinib/cytarabine for the first-line treatment of early Philadelphia chromosome–positive chronic myeloid leukemia: results of a randomized clinical trial of the Mexican collaborative leukemia group. Clin Leukemia. (2008) 2:128–32. 10.3816/CLK.2008.n.016

[42] Deenik W Janssen JJ van der Holt B Verhoef GE Smit WM Kersten MJ. Efficacy of escalated imatinib combined with cytarabine in newly diagnosed patients with chronic myeloid leukemia. Haematologica. (2010) 95:914–21. 10.3324/haematol.2009.016766 20015886 PMC2878788

[43] Cortes J Lipton JH Rea D Digumarti R Chuah C Nanda N. Phase 2 study of subcutaneous omacetaxine mepesuccinate after TKI failure in patients with chronic-phase CML with T315I mutation. Blood. (2012) 120:2573–80. blood-2012-03-415307 22896000 10.1182/blood-2012-03-415307 PMC4916583

[44] Rosshandler Y Shen AQ Cortes J Khoury HJ. Omacetaxine mepesuccinate for chronic myeloid leukemia. Expert Rev Hematol. (2016) 9:419–24. 10.1586/17474086.2016.1151351 26853281

[45] Wylie AA Schoepfer J Jahnke W Cowan-Jacob SW Loo A Furet P. The allosteric inhibitor ABL001 enables dual targeting of BCR-ABL1. Nature. (2017) 543:733–7. 10.1038/nature21702 28329763

[46] Hughes TP Goh YT Ottmann OG Minami H Rea D Lang F Expanded phase 1 study of ABL001, a potent, allosteric inhibitor of BCR-ABL, reveals significant and durable responses in patients with CML-chronic phase with failure of prior TKI therapy. Blood. (2016) 128:625 Available online at: http://www.bloodjournal.org/content/128/22/62527297793

[47] Massimino M Stella S Tirro E Romano C Pennisi MS Puma A. Non ABL-directed inhibitors as alternative treatment strategies for chronic myeloid leukemia. Mol Cancer. (2018) 17:56. 10.1186/s12943-018-0805-1 29455672 PMC5817805

[48] Brauer KM Werth D von Schwarzenberg K Bringmann A Kanz L Grunebach F. BCR-ABL activity is critical for the immunogenicity of chronic myelogenous leukemia cells. Cancer Res. (2007) 67:5489–97. 10.1158/0008-5472.CAN-07-0302 17545631

[49] Bacigalupo A Soracco M Vassallo F Abate M Van Lint MT Gualandi F. Donor lymphocyte infusions (DLI) in patients with chronic myeloid leukemia following allogeneic bone marrow transplantation. Bone Marrow Transplant. (1997) 19:927–32. 10.1038/sj.bmt.1700762 9156268

[50] Christiansson L Soderlund S Svensson E Mustjoki S Bengtsson M Simonsson B. Increased level of myeloid-derived suppressor cells, programmed death receptor ligand 1/programmed death receptor 1, and soluble CD25 in Sokal high risk chronic myeloid leukemia. PLoS ONE. (2013) 8:e55818. 10.1371/journal.pone.0055818 23383287 PMC3561335

[51] Prost S Relouzat F Spentchian M Ouzegdouh Y Saliba J Massonnet G. Erosion of the chronic myeloid leukaemia stem cell pool by PPARgamma agonists. Nature. (2015) 525:380–3. 10.1038/nature15248 26331539

[52] Herrmann H Sadovnik I Cerny-Reiterer S Rulicke T Stefanzl G Willmann M. Dipeptidylpeptidase IV (CD26) defines leukemic stem cells (LSC) in chronic myeloid leukemia. Blood. (2014) 123:3951–62. 10.1182/blood-2013-10-536078 24778155

